# Sexual habits and chemsex use in a monographic consultation for sexually transmitted infections (STI)

**DOI:** 10.1192/j.eurpsy.2025.2309

**Published:** 2025-08-26

**Authors:** C. Herranz Serfaty, S. Moreno Guillen, M. Sánchez, A. Muñoz San José, M. E. Sánchez-Escalonilla Relea, M. Viana Perez, Á. Esquembre García

**Affiliations:** 1 Psychiatry, Hospital Universitario La Paz; 2 Infectious diseases Deparment, Hospital Universitario Ramón y Cajal, MADRID, Spain

## Abstract

**Introduction:**

The use of recreational drugs for sexual intercourse (*chemsex*) is a widespread practice in certain subgroups of the population in recent years. There is documentation of chemsex in men who have sex with men (MSM) but less data on its use in other populations (men who have sex with women, women, transgender women, transgender men) and its effects on mental health.

**Objectives:**

Evaluate the use of chemsex in the population attended in a sexually transmitted infection (STI) consultation.

**Methods:**

To evaluate the use of chemsex in the population attended in a sexually transmitted infection (STI) consultation, a survey was carried out on all patients who attended the STI consultation of the U. Ramón y Cajal Hospital between January and April 2022. The degree of anxiety and depression was assessed using the HADS scale.

**Results:**

A total of 148 surveys were distributed, with 82 being completed. Among those surveyed, 56% had used drugs at some time in their lives, the vast majority of times associated with sexual relations. The most consumed drugs were alcohol, cannabis and poppers. There were no differences between MSM and non-MSM in this consumption (p= 0.073), but there were in the substances consumed: MSM consumed more gamma hydroxy-butyrate acid (GHB/GBL) (p= 0.031), mephedrone (p= 0.031) and poppers (p= 0.019). Using the HADS Scale, 34 patients suffered from anxious (41%) and 11 depressive symptoms (13%), with no significant differences between MSM and non-MSM.

**Conclusions:**

*Chemsex* is a frequent phenomenon among patients attending in an STI consultation, both in the MSM and non-MSM population. It negatively impacts mental health, being associated with anxiety and depression. It is necessary to improve information to reduce drug use in this context.

**Disclosure of Interest:**

None Declared

